# Tumor-derived extracellular vesicles: reliable tools for Cancer diagnosis and clinical applications

**DOI:** 10.1186/s12964-019-0390-y

**Published:** 2019-07-10

**Authors:** Reza Rahbarghazi, Nasrollah Jabbari, Neda Abbaspour Sani, Rahim Asghari, Leila Salimi, Sadegh Asghari Kalashani, Maryam Feghhi, Tahereh Etemadi, Elinaz Akbariazar, Mahmoud Mahmoudi, Jafar Rezaie

**Affiliations:** 1 Stem Cell Research Center, Tabriz University of Medical Sciences, Tabriz, Iran; 2 Department of Applied Cell Sciences, Faculty of Advanced Medical Sciences, Tabriz University of Medical Sciences, Tabriz, Iran; 3 Solid Tumor Research Center, Cellular and Molecular Medicine Institute, Urmia University of Medical Sciences, Shafa St, Ershad Blvd., P.O. BoX: 1138, Urmia, 57147 Iran; 4 Department of Medical Physics and Imaging, Urmia University of Medical Sciences, Urmia, Iran; 5 Department of Oncology, Imam Khomeini hospital, Urmia University of Medical Sciences, Urmia, Iran; 6 Department of Medical Laboratory Sciences, Imam Khomeini hospital, Urmia University of Medical Sciences, Urmia, Iran; 7 Department of biology, faculty of sciences, Arak University, Arak, Iran; 8 Department of Genetic, Urmia University of Medical Sciences, Urmia, Iran; 9 Department of Medical Parasitology, Urmia University of Medical Sciences, Urmia, Iran

**Keywords:** Extracellular vesicles, Cancer, miRNA, Biomarker, Drug delivery

## Abstract

**Background:**

Studies have recently revealed that almost every type of cells including tumor cells abundantly release small vesicles known as extracellular vesicles (EVs) into the extracellular milieu. EVs carry a repertoire of biological molecules including nucleic acids, proteins, lipids, and carbohydrates and transport their cargo between cells in the vicinity as well as distantly located cells and hence act as messengers of intercellular communication. In this review, we aimed to discuss the tumor-derived exosome biology and the pivotal roles of exosomes in cancer diagnosis and treatment.

**Methods:**

In the present review study, the authors studied several articles over the past two decades published on the kinetics of EVs in tumor environment as well as on the application of these vesicles in cancer diagnosis and therapy.

**Results:**

A growing body of evidence indicates that nucleic acids such as microRNAs (miRNAs) transferring by EVs participate to create a conducive tumor environment. As EV-associated miRNAs are tissue-specific and present in most biological fluids, they hold great potential for clinical application in cancer early diagnosis, prognosis, and treatment response. Furthermore, exosomes can serve as drug delivery vehicles transferring miRNAs as well as therapeutic agents to target cells. These nano-vesicles exhibit ideal properties in comparison with the synthetic carriers that attracted scientist’s attention in the field of nanotechnology medicine. Scientists have employed different strategies to build exosomes-based drug delivery system. In general, two methods (direct engineering and indirect engineering) are being utilized to produce artificial exosomes. Para-clinical data have confirmed the beneficial effects of engineering exosomes in cancer therapy.

**Conclusion:**

Exosomal miRNAs hold great promise for clinical application in early diagnosis and treatment of cancers. In addition, in spite of enthusiastic results obtained by engineered exosomes, however, there is an increasing concern over the use of optimal methods for engineering exosomes and the safety of engineered exosomes in clinical trials is still unclear.

## Background

Cancer, the second reason for mortality worldwide, has grown to be a global health issue during past decades [1]. In a tumor environment, the tumor cells exhibit unique characteristics such as their co-option with surrounding cells and soluble factors, uncontrolled growth, immune evasion and resistance to therapies [2]. Early identification of tumor is a pivotal factor in cancer therapy that improves survival and quality of a patient’s life [3]. There are possible challenges in the invasive biopsy technique, which biopsies samples may don’t represent exact information about heterogeneous tumor tissue. Additionally, it may cause stimulation in tumor growth rate and metastasis [4]. Alternatively, liquid biopsy is a simple and non-invasive way to inform clinicians about the tumor progression through a bio-fluid sampling such as blood sampling [5]. Recent studies suggested that tumor condition could be monitored by cell-derived nanoparticles named EVs which carry bio-molecules including mRNA, miRNAs, DNA, proteins, and lipids [6]; as well as long-non coding RNAs [7] contribute to the intercellular communication. It has been shown that EVs contain the information of tissue of their origin [8]. Researchers have demonstrated that EVs including exosomes provide a critical and sensitive source of biomarkers in almost pathological condition [9–11]. Exosomes, a subtype of extracellular vesicles (EVs), are cell-derived biopolymers in the range of 30–120 nm in diameter, which have unique structural, biochemical and mechanical properties [12]. These vesicles encompassing bio-molecules including mRNA, miRNAs, DNA, proteins, and lipids; contribute to the intercellular communication. In tumor cells, exosome biogenesis, and loading mechanisms is a complex and regulated process, and many different molecules passively and/or actively involved in to form exosomes [13]. As exosomes distribute through bio-fluids, they are more accessible through simple liquid sampling. Thus, by analyzing the exosomal miRNAs signature, scientists are capable of understanding secreting cell status and eventually tumor condition [14]. Furthermore, due to shuttling various biomolecules, there is an idea that exosomes can be used for cancer therapy by delivering therapeutic agents [15]. Exosomes pose such favorable characteristics that make them superior to traditional nano-delivery modules. For example, exosomes are cell-derived particles, therefore, exhibit more safety and stable property than other delivery compounds such as liposomes [16]. Besides, they specifically deliver cargoes to the distant target sites, even they pass through the blood-brain barrier [16]. In this regard, laboratories have examined the possible therapeutic potential of exosomes by sorting miRNAs and therapeutic compounds into exosomes in vitro and in animal models [17, 18]. Various strategies have been employed for loading therapeutic agents into exosomes and authors tracked efficacy and specificity of exosome-based drug delivery system in the in vitro and in vivo models [19, 20]. In this review, we discuss the recent progress in the exosome kinetic, especially crucial roles of exosomal miRNAs in the tumor microenvironment and their application in cancer diagnosis. In addition, we review the last knowledge about strategies used to engineer and form optional exosomes and also explain examples of the therapeutic agents sorted into exosomes.

### Biology of extracellular vesicles

**Fig. 1:**
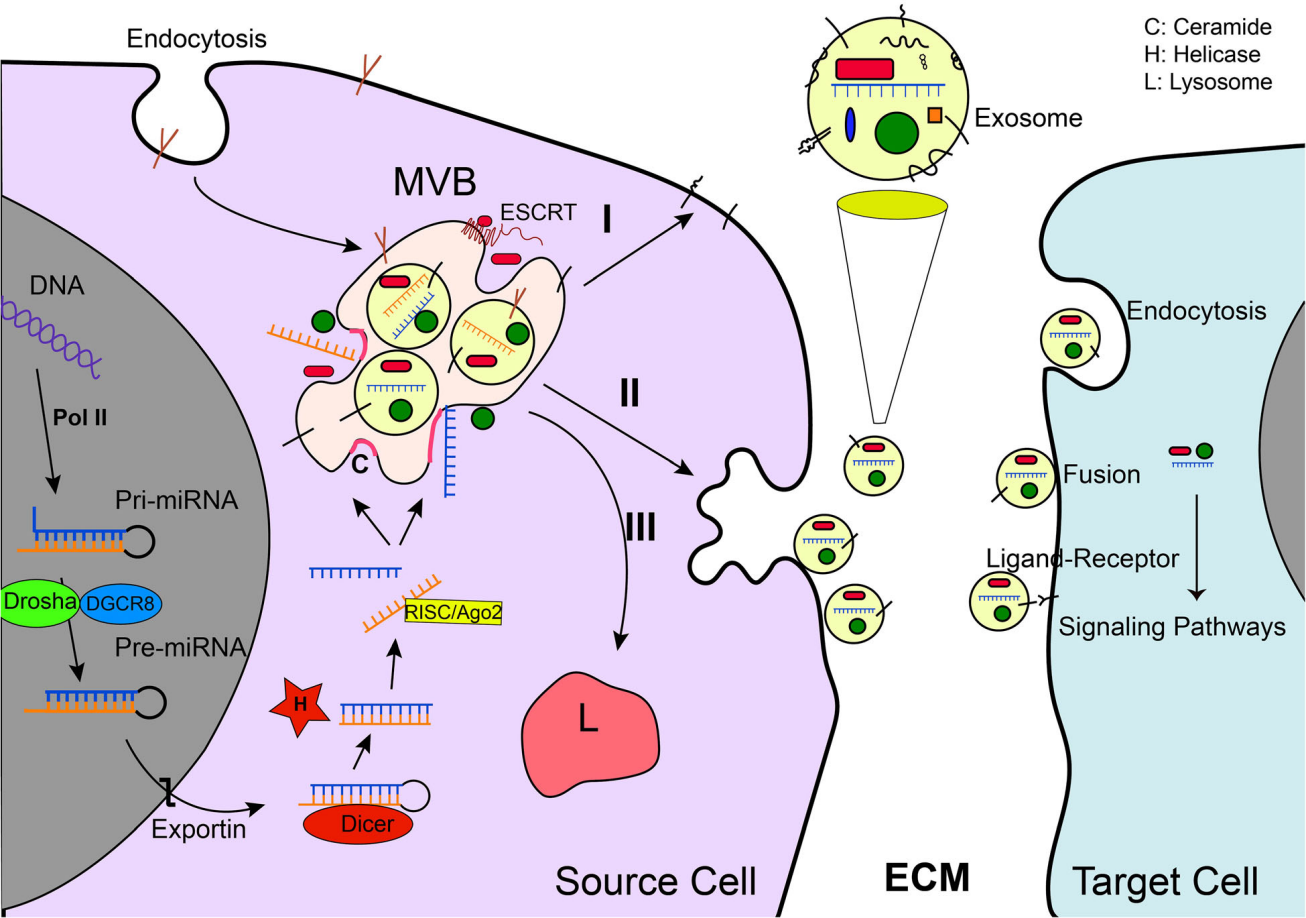
The mechanism of exosomal miRNAs loading. In the nucleus, Pol-II enzyme transcribes primary miRNAs (pri-miRNA) from miRNA-related genes. DGCR8 and Drosha structure catalyzes pri-RNAs into pre-miRNAs form, which is transferred toward cytoplasm employing exportin-5 protein. At the cytoplasmic level, the pre-miRNAs are trimmed into double-stranded miRNAs through the Dicer complex action. Then Helices, a splicing enzyme, generates mature miRNAs which harbors single-stranded. In the final step, MVBs (exosomes) capture mature miRNAs via four possible mechanisms including: 3′ miRNA sequence-dependent pathway, nSMase2-dependent pathway, the miRNAISC-based pathway, and sumoylated hnRNPs-related pathway. Other biological materials are sorted into exosomes through endocytosis, Golgi apparatus, different protein complexes, and ESCRT machinery randomly or/and preferentially. Of note, after exosome maturation, MVB could back fuse with the PM in which decorate the mother cell PM with specific receptors (I). Another fate is that MVB combine to the PM and exports its cargoes to the ECM (pathway II). Scientists believe that MVB may select degradation pathway where it combine with lysosomes (pathway III) and its content is degraded. Different proteins such as Rab-GTPase and SNAREs contribute in intracellular MVB trafficking and fusion. Three possible mechanisms were proposed by which exosomes can alter target cell function, I: endocytotic pathway; II: ligand-receptor interaction; III: direct fusion

Recent progress in molecular medicine described the key roles of EVs in cell biology. Especially, EVs are introduced as intercellular messenger in physiological and pathological conditions [13]. They encompass a large number of bio-martial such as RNAs, DNA strands, proteins, and also lipids [21]. According to evidence, three subpopulations of EVs were detected based upon the biogenesis mechanism and the size of vesicles diameter [22]. Exosomes, the 30–120 nm member of EVs, releasing from almost different types of mammalian cells through the endocytotic pathway [23]. Interestingly, recent scrutiny on exosomes purification has identified two distinct subpopulations of exosomes according to their size and compositions [24]. Exosomes distribute both in cell culture medium and body bio-fluids including blood, CSF, urine, and breast milk [25]*.* Exosome biogenesis is an intracellular complex mechanism that various types of molecules including endosomal sorting complex required for transport (ESCRT) assembly, lipid molecules, accessory proteins, Rab-GTPase family, and soluble NSF attachment protein receptors (SNAREs) proteins contribute to regulating exosome formation, loading, and releasing [20, 26] (Fig. 1). Beside transferring various molecules, exosomes have been shown to express the conventional markers including CD63, CD82, CD9, CD81, ALIX, and also TSG101 [23]. Multivesicular bodies (MVBs) are late endosome compartments located in the cytoplasm, assigned to generate exosomes. Previous experiments have confirmed that mature MVBs have three intracellular fates (Fig. 1); secretory, lysosomal, and back fusions fates [27]. In the secretory pathway, MVBs could combine with the plasma membrane (PM) and unload vesicles named exosomes into the extracellular matrix (ECM). Once exosomes distributed to the ECM, they try to target cells in proximity or in faraway. Scientists supposed three possible ways that exosomes contribute to affect recipient cell function (Fig. 1); (i) internalization; (ii) receptor-ligand interaction; (iii) direct fusion [27, 28]. Internalization pathway is a traditional way to import exosomes, where exosomal cargoes contribute to affect signaling pathways in recipient cells. Another way is ligand-receptor interaction, in this way exosomal surface molecules interact with molecules located on the PM of target cells [29]. In direct-type fusion, these vesicles may well directly fuse with the PM of recipient cells through engaging common fusion-related molecules such as SNAREs. Emerging evidence for further uptake ways has been reported, for example, protease located in the ECM may be responsible of activation of exosomal proteins/enzymes that in turn, they contribute to interact with target cells receptors as a ligand, then induce receptor-related downstream signaling [30]. Microvesicles (MVs), 100–1000 nm vesicles, a subtype of EVs, are released from various cells including plates and endothelial cells (ECs). There is a consensus that MVs are made by the mechanism that the PM abscises encompassing cytoplasm materials. These vesicles are irregular in shape, heterogeneous in size and different in contents based on maternal cell physiological status [31, 32]. The largest subclass of EVs (1–6 μm in diameter) is apoptotic bodies (ABs), deriving from cells undergoing apoptosis [33]. There appears to be a consensus of opinion that ABs formation occurred when caspase-3 activates a kinase, in turn, that kinase phosphorylates myosin light chain and initiates splitting cells up into segmentations [34]. ABs harbor cytoplasmic components, therefore, they may mediate communication signaling among target cells and progress adverse effects in several diseases [35]. Recent trends in the kinetic of EVs contributed to the launch of the International Society for Extracellular Vesicles (ISEV, www.isev.org), which is a worldwide society of leading EVs, exosomes, and MVs researchers. ISEV internationally authorized to advance EVs research and instruction including refreshes the terminology, categorization, isolation, characterization, and functional analysis. In addition, with more than 1,000 members, ISEV organization all around interfaces top scientists at its annual meeting, workshops and different occasions. To date, there are databases such as ExoCarta (www.exocarta.org), Vesiclepedia (www.microvesicles.org), and EVpedia (www.evpedia.info) assembling exosomal contents for the researchers. This fact globally shows the significance of EVs in biological systems and also in the field of disease management. Nowadays, scientists interested in the field of clinical application of EVs in various diseases such as cancers. In the case of cancers, the interesting aspects of EVs are the diagnosis, prognosis, and therapeutic potentials.

### Exosome cargoes

Exosomes are spheroid particles composed of a bilayer lipid membrane structure that decorated themselves surface with various ligands and receptors harbored from parental cells. In addition, their lumen contains various biomolecules such as nucleic acids, carbohydrates, and proteins [36]. Recent progress in the field of exosome isolation and characterization led to considerable interest in the discovery of exosomes cargoes because exosomal molecular cargoes can increase our knowledge about underlining mechanisms involved in biogenesis and function of exosomes; especially, cargoes may serve as biomarkers and therapeutic agents for various disease diagnosis and treatment [37]. Nevertheless, there is evidence that suggests exosomes from different source exhibit even common molecules. For instance, as exosomes are manufactured from the endocytotic pathway, they inherit endosomal components such as Alix and Tsg101 molecules. Other molecules for example tetraspanins (CD63, CD81, and CD9), intracellular trafficking molecules (Rab-GTPase family and SNAREs), membrane molecules (integrins, adhesion molecule 1 (ICAM-1)), cytoskeletal components (tubulin, actin, and annexins) are traditionally present in almost exosomes [38, 39]. As well, previous studies have reported that common lipids for instance phosphatidylethanolamine, diacylglyceride, ceramides, phosphatidylserine, also-bisphospatidic acid, cholesterol, and sphingomyelin are present in exosomes [40]. In addition, for the first time, the existence of nucleic acids in cargoes of exosomes has been detected in exosomes from mast cells [41]. After that, growing evidence confirmed the existence of different kind of the mRNAs and miRNAs in various exosomes. It seems that a complex sorting mechanism is involved in the exosomal loading process rather than a random process [42]. Of note, cells preferentially engage complicated mechanisms to sort specific nucleic acids to the exosomal secretory pathway [43]. Interestingly, it has been demonstrated that in the pathological condition exosomes cargoes are different from in the normal one. Thus, parental cell status may be disclosed by analyzing the composition of isolated exosomes and discrimination between the types of components can be used as a pivotal biomarker of disease [44]**.** Stress condition is another factor that influences exosomes cargoes. For example, there is a growing body of evidence that ionizing radiation [45] and hypoxia [46] not only increase exosome secretion rate but also alter exosome cargoes. More recently, a database (http://bioinfo.life.hust.edu.cn) has established to present 462 small RNA sequencing samples of EVs from 17 sources which show miRNA expression profile in different EVs [47]. Another informative database, Exocarta (http://www.exocarta.org), the globally exosomal database, categorized 1,639 mRNAs, 4764 miRNAs, 563 proteins, and 194 lipids based on organisms [48]. Similarly, Vesiclepedia (http://www.microvesicles.org) is an exosome-related database that prepared a large number of data about exosomes cargoes from various studies. This web has collected 1254 EVs studies and categorized approximately 349988 proteins, 38146 various RNAs, 639 lipids [49]. Collectively, these facts show that exosomes act as bio-container transferring a large number of biomolecules and different cells release various exosomes with distinct contents.

### Exosomal miRNA loading

It seems that the exosome loading process is complex and mediated by different biomolecules located on MVBs membrane [26] and there is a little information about underlying mechanisms [22]. However, it has been uncovered that the ESCRT complex contributes to packaging-specific molecules into exosomes. In this scenario, different biological components may be trapped constitutively or randomly during exosomes biogenesis [13]. As it is known, small RNAs such as miRNAs, small non-coding RNAs, be composed of 22 nucleotides, expressed in eukaryotes and even in a number of viruses [50]. Based on previous studies, miRNAs are generated in a precursor form called pre-miRNAs (Fig. 1). This miRNA interconnected with the performing compound including argonaute2 (Ago2), human embryonic lethal abnormal visual (ELAV proteins or human antigen R (HuR), Dicer, and various RNA-binding proteins (RBPs) which make mature miRNAs in the cytoplasm. Finally, mature miRNAs contribute to blockage RNA translation; therefore, regulate mRNAs fate (Fig. 1). In fact, studies of miRNAs function indicate that miRNAs inhibit mRNA molecules through three possible mechanisms; I: segmentation of the mRNA strands, II: destabilization of the mRNAs, and III: inhibition of the mRNA- ribosomes interaction [51, 52]. Study of exosome biogenesis process shows the importance of sphingomyelinase 2 (nSMase2) in the loading of exosomal miRNAs [53]. A possible explanation is that nSMase2 catalyzes the formation of ceramide, which promotes exosome formation and miRNA trapping. In support, it has been reported that the interrupting of nSMAse2 activity blocks exosomes secretion [54]. Besides, exosomal packaging of miRNAs is regulated through a sequence-dependent mechanism and mediated by chaperone proteins [43]. A work conducted by Villarroya-Beltri et al. [55] declared that the hnRNPA2B1 protein, associated with emerging pre-mRNAs, packaging pre-mRNAs into hnRNP particles, participate to target specific miRNAs into exosomes. Interestingly, 3′ end sequences of miRNAs is a landmark for effector proteins to determine whether miRNAs are reserved in the intracellular pool or engaged for export via exosomes. For example, it was approved that presence of 1, 2 or 3 adenosine or uridine nucleotides in the 3′ end of miRNA may direct miRNA to exosomes. Interestingly, poly-U miRNAs are packaged into exosomes while poly-A miRNAs probably remain in the cytoplasm [56]. Moreover, in comparison to the cell, exosomes transfer low volume of ribosomal RNA (rRNA) such as 28 s and 18 s rRNA and RNAs content of exosomes differ to cellular content [41]. A large of evidence supports the fact that RBPs contribute to sorting special RNAs into exosomes. For example, RNAs in association with Ago2, a type of RBPs, and high-density lipoprotein components were discovered in an extracellular milieu in the form of EVs-linked or free of EVs. It is reasonable that RBPs may protect RNAs in extracellular space and inside exosomes [57, 58]. A work conducted by Steatello and colleagues identified 30 RBPs in exosomes that contribute to forming RBP-RNA complexes. Notably, authors by doing functional experiments on 6 RBPs genes showed that MVP is a key member of RBRs that regulate sorting process of RNAs into exosomes and protect them inside exosomes in ECM [59]. Alike, tumor-derived exosomes potentially transfer miRNAs linked to the RISC-Loading Complex (RLC). In addition, complex molecules such as Dicer, TRBP, AGO2, and also pre-miRNA, were associated with exosomes of cancer cells. Besides delivering mature miRNAs, it seems that exosomes contribute to the development of precursor pre-miRNA into mature form by a cell-free mechanism in the cytoplasm of recipient cells [60]. A work on colorectal cancer cells showed that the exosomal loading mechanism is a KRAS-dependent and KRAS-mutant cells abundantly release miRNA-100 while normal KRAS cells sort a high volume of miRNA-100 to the exosomes lumens incorporation with nSMAse [61]. Recent evidence suggests that KRAS/ MEK/ERK signaling pathway may abolish the packaging of AGO2-linked miRNAs into exosomes [62]. These findings indicate that exosomal miRNA loading is a tightly regulated mechanism. However, most of the exosome-based studies have only described exosomal miRNAs profiling of different cancer cells and there is a little knowledge about miRNAs loading mechanisms [63]. Furthermore, by which mechanisms RNA-protein complexes are selected by MVBs and how RNA molecules preserved in exosomes still remain elusive. Noticeably, several attempts have been made to assay effect of environmental condition on exosomes loading rate and composition. For instance, Jung and the co-workers showed that hypoxia altered breast cancer cells derived exosomes miRNAs content, so that miRNA-210 was abundantly burdened into those exosomes [64]. Ionizing radiation is another stress factor that influences exosome formation and loading through p53/ tumor suppressor activated pathway 6 (TSAP6) signaling pathway [45]. In this sense, the p53/TSAP6 axis has been shown to mediate exosomal miRNAs loading process following DNA damage. In an analysis of exosomal miRNAs content from different tumor cells lines, Langevin et al. found that there was noteworthy dissimilarity in the miRNAs profile of exosomes among tumor cells and normal ones [65]. Thus, at least, the miRNA loading system in tumor cells selectively introduces specific miRNAs to exosomes generator compartments based on cell condition [34]. The central questions, in this case, are: Do different cells use the same mechanism to load miRNAs into exosomes? And; in different conditions, which miRNA loading mechanism is involved?

### Role of exosomal miRNAs in cancer biology

Enriched in exosomes, miRNAs transmitted into target cell cytoplasm and participate to regulate mRNAs expression and cell function of target cell [41]. Evidently, exosomal miRNAs attracted scientific attentions have pivotal roles in spreading the adverse effects of tumors [66]. Cancer cells release more exosomes to promote many aspects of tumors malignity including proliferation, invasion, metastasis, angiogenesis, and immunosuppression [67]. In the case of proliferation, in human nasopharynx cancer, tumor-released exosomes actively transfer onco-miRNAs such as miRNA-106a-5p, miRNA-891a, miRNA-24-3p, and miRNA-20a-5p that promote cell proliferation through inhibition of MARK1 protein signaling pathway [68]. Conversely, in lung carcinoma cell lines, 95C and 95D cell derived exosomes deliver miRNA-302b to target cells and cause cell growth inhibition by the TGFβRII/ERK signaling pathway [69]. Kogure and colleague declared that exosomal miRNA-584 is capable of accelerating hepatocellular cancer cells proliferation [70]. Several researchers have emphasized that oncogenic exosomal miRNAs are capable of inducing metastasis and aggressive mass out of the tumor location. For example, it was previously documented that tumor-derived exosomes by miRNA-29a and miRNA-21 contribute to tumor cell proliferation and invasion. It seems that activation of NF-κB by toll-like receptors (TRL) downstream signaling in immune cells led to producing cytokines from immune cells that amplify tumor cell division and metastatic rate [71]. It was represented that tumor-released miRNAs act as a ligand for TLR family, human TLR8, and murine *TLR7* in immune cells that promote pro-metastatic inflammatory factors and eventually results in tumor cell movement and aggressiveness [72]. In addition, miRNA-105 loaded exosomes from cancerous cells stimulating invasion to the brain and respiratory system; act as a ZO-1 inhibition agent in ECs and facilitate cell migration [73]. In this regard, miRNA-21 bearing exosomes stimulate the invasion and relocation of esophageal tumor cells via activating PDCD4 / JNK axis in recipient cells [74]. Furthermore, in another study, exosomal miRNA-122 from breast tumor cells contributes to rearrange glucose metabolism, in turn, promotes metastasis niche formation [75]. In addition, rat BSp73ASML cancerous cells contain onco-miRNAs such as miRNA-494 and miRNA-542-3p that regulate the formation of cadherin-17 and matrix metalloproteinase (MMPs) hence promote the formation of a pre-metastatic niche in recipient host tissues [76]. Recently, investigators have examined the effects of tumor-derived exosomal miRNAs on the angiogenesis. Angiogenesis, raising new capillary from preexistent vessels, is a critical key in tumor expansion stimulated by tumor hypoxic environment [77]. The potential relationship between exosomal miRNAs and angiogenesis has arisen from the observation that hypoxic A549 lung tumor cells secret miRNA-494 bearing exosomes that delivered to neighboring ECs and encouraged the angiogenic potential through inducing Akt/eNOS axis and block of PTEN protein [78]. Moreover, miRNA-21 released by exosomes from lung tumor cells by way of STAT3 signaling and VEGF generation promotes angiogenesis in non-tumor lung cells [74]. Chronic lymphocytic leukemia cells release exosomal miRNAs targeting mesenchymal stem cell (MSCs) and ECs that leading to force cells to produce pro-angiogenic molecules [79]. Additionally, exosomes released from prostate cancer cells can participate to weaken the ECM and induce angiogenesis. These exosomes contain miRNA-21-5p, miRNA-139-5p mediating the activation of MMP2,-9,-13 enzymes [80]. By administration of exosomal miRNA-92a derived from K562 cells, Umezu et al. elucidated that angiogenic potential of ECs such as immigration and tubulogenesis were enhanced [81]. Thereafter, contrariwise, miRNA-92a has been shown to have antiangiogenic roles. In this way, when it was secreted within MSCs-derived exosomes, it acts as an anti-angiogenic factor [82]. Alike, the key role of miRNA-9 in angiogenesis is controversial. For example, exosome isolated from engineered nasopharyngeal carcinoma cells inhibits the angiogenic ability of endothelial cells by targeting MDK and PDK1/AKT pathways [83]. A work by Zhuang et al. showed that miRNA-9 has pro-angiogenic activity through reducing expression of SOCS5 gene and promoting the JAK-STAT axis function, which supports ECs migration and tumor angiogenesis [84]. In line with this, Qu et al. detailed that high level of miRNA-9 can differentiate MC3T3-E1 cells to osteoblast cells and increases angiogenesis via AMPK–dependent pathway [85]. Another potential role of exosomal miRNAs is immunosuppression by which reprogram the cells of the immune system, for instance, natural killer (NK) cells and dendritic cells (DCs) [86]. DCs, a key sub-population of immune cells, encourage immunity or make immune- tolerance according to the state of activation [87]. In this regard, Que. and co-workers demonstrated that exosomal miRNA-212-3p from pancreatic cancer cells degrades Regulatory factor X-associated protein (RFXAP) mRNAs in DCs, leading to immune tolerance through minimizing of MHC II molecules [86]. EVs miRNAs also play as a key regulator of NK cells. For example, MVs released from hypoxic tumor cells adversely affect NK cells function via delivering miRNA-23a and TGF-β [88]. Interestingly, Yin et al. pointed out the exosomal miRNA-214 induces immunological tolerance response in CD4^+^ T cells. They also confirmed that tumor-induced immune tolerance was reversed when exosomes purified from human embryonic kidney cell line 293 (HEK293) delivered antisense copy of miRNA-214 to T helper cells [89]. Thus, preliminary data suggest that tumor-derived exosomal miRNAs participate to inducing tumor tolerance of the immune system. In conclusion, tumor-derived exosomes have pivotal roles in cancer progression; especially their miRNA cargoes contribute to manipulating transcriptome pool of target cells. It seems that discovery in the field of exosomal miRNAs biology could uncover the underlying mechanisms promoting the aggressive feature of tumors.

### Exosomal miRNAs as a tumor biomarker

In the new global oncology, tumor-derived exosomes have become a central candidate for promoting tumor survival, invasion, metastasis, angiogenesis, and immunomodulation through transmitting onco-molecules including miRNAs, DNA, proteins, and lipids [67]. As miRNAs bearing exosomes can be distributed to bio-fluids, therefore, liquid-biopsy from urine, plasma, and CSF is a non-invasive method for obtaining exact information about tumor environment/status [63]. Firstly, Taylor and colleagues reported that serum biomarkers such as miRNA-214, miRNA-205, miRNA-203, miRNA-141, miRNA-200 a,-b,-c, and miRNA-21 are also rich in sera-derived exosomes of patients suffering from ovarian tumors (Table 1) [102]. In lung adenocarcinoma patients, miRNAs analysis showed a significant correspondence between tumor cell miRNAs and circulatory exosomal miRNAs expression pattern. Thus, these miRNAs may be considered as a biomarker of lung cancer. Moreover, it was revealed that total exosomes and miRNAs were enhanced in patients in comparison to the healthy group [103]. Similarly, using wide-range microRNAs analysis, Cazzoli and et al. declared that the exosomal miRNAs including miRNA-200-5p, miRNA-378a, miRNA-379, and miRNA139-5p could serve as biomarkers of lung adenocarcinoma [8]. In the context of breast tumors, biomarker application of exosomal miRNAs was assayed using cancerous cell lines including MCF-7 carcinoma cells and also MDA-MB-231 in vitro*.* For example, Kruger et al., discovered that MCF-7 cells release exosomes enriched with miRNAs such as miRNA494, miRNA-198, miRNA34a, and miRNA-26a, whereas exosomal miRNA-328– 5p, miRNA-149– 3p, and miRNA-130a were available in the epithelial cancerous cells; MDA-MB-231 cell line [104]. In triple negative breast malignancy individuals, circulatory levels of exosomal miRNA-373 are considerably up-regulated, thus it may be used as cancer biomarker [105]. Up-regulation of exosomal miRNA-19a in the bloodstream of colorectal cancer (CRC) subjects were recorded and interestingly introduced as a relapse biomarker of CRC [106]. Furthermore, the expression pattern of the exosomal miRNA-17-92a family in the circulatory system was significantly correlated to the CRC relapse rate. In the last few years, some laboratories used urine exosomal miRNA as prostate and bladder cancers biomarkers, so that high level of exosomal miRNA-21 [92] and miRNA-21-5p [107] were considered as an indication of prostate and bladder cancer development respectively. We have shown in Table 1 exosomal miRNAs that may use as cancer diagnostic biomarkers in different cancers.

**Table 1:** Possible exosomal miRNAs as tumor biomarker

Cancer	miRNAs considered as biomarkers	Source	Reference
Acute myeloid leukemia	miR-150, miR-1246, miR-155	Serum	[90]
Bladder	miR-15b, miR-24, miR-135b, miR-1224-3p	Urine	[91]
	miR-21, miR-4454	Urine	[92]
Breast	miR-200a, miR-200c, miR-205	Serum	[93]
	miR-10, miR-21, miR-182, miR-373, miR-1246	Serum	[94]
Cervical	miR-21, miR-146a	Cervical lavages	[95]
Colorectal	miR-23a, miR-1229, miR-1246, miR-let-7a, miR-150, miR-21, miR-223	Serum	[96]
	let7a, miR-21, miR-192, miR-221	Serum	[97]
Esophageal	miRNA-21	Serum	[98]
Glioma	miR-320, miR-574-3p	Serum	[99]
	miR-21	CSF	[100]
Lung	miR-155,miR-17-3p, miR-205, miR-21, miR-106a, miR-146, miR-191, miR-192, miR-212, miR-214 miR-203, miR-210	Plasma and Bronchoalveolar	[101]

### Exosome-based miRNA delivery system

**Fig. 2:**
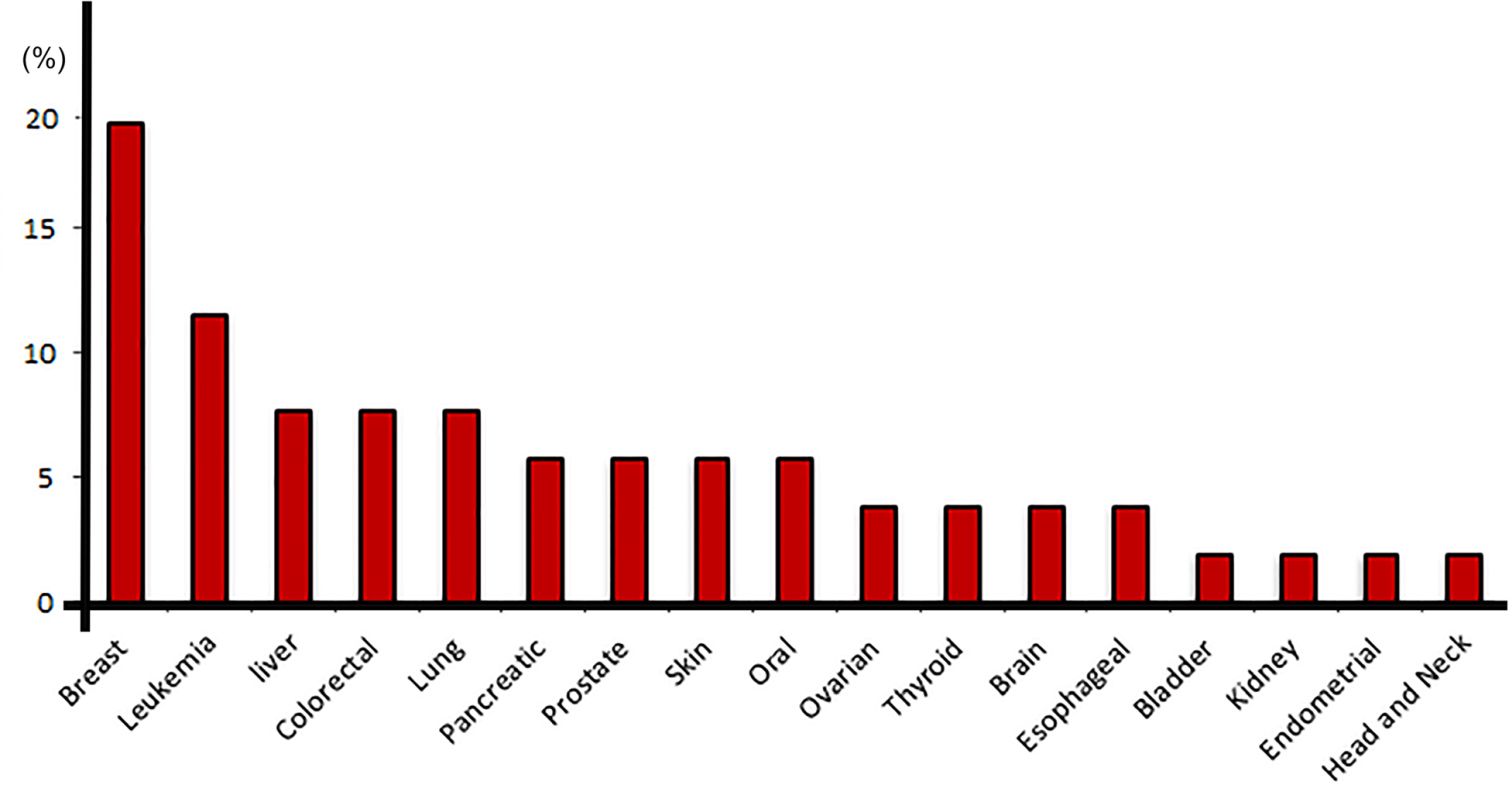
A diagram of the miRNAs application in clinical trials depended on the available data up to May 2019. Data show the majority of miRNA-related clinical trial belongs to breast cancer. Diseases are presented based on the percentage of the clinical trials deal with miRNAs

A recent development in the cancer therapy led to design the miRNA therapy systems, which engage tumor- suppressor miRNAs for shrinkage tumor mass [108]. In this regard, exosomes as a bio-container of non-coding RNA are taken into consideration [109]. Recent reports have revealed that exosomal miRNAs are the imperative effectors of cells in the biological environment [41, 110]. As miRNAs actively regulate cell function, it is sensible to recommend that engineered exosomal miRNAs are a useful tool in the management of diseases [111, 112]. Kosaka and coworkers showed that intra-tumor administration of miRNA-143-enriched conditioned medium (CM) to nu/nu mice with grafted prostate tumor caused the cancer cell shrinkage. Besides, the mRNA level of KRAS and ERK5 as target genes of miRNA-143 decreased following injection of miRNA-143- transduced CMs. Conversely, the growth rate of normal cells was not suppressed by exogenously-transduced miRNA-143. Thus, they concluded that exosome-based miRNAs therapy may serve as a specific tool without non-targeting effect [113]. Lou et al. found that MSCs-derived exosomes as miRNA-122 vehicle increase hepatocellular carcinoma chemosensitivity. In this vein, use of chemotherapeutic agents along with the delivery of miRNA-122 via MSC–derived exosomes deeply caused fall in the cell cycle at G0/G1 level and increased cell apoptosis rate, what signifies a hopeful approach for hepatocellular carcinoma treatment [114]. Moreover, exosomes exogenously enriched with siRNAs to inhibit diseases, for example, it has recently been shown that exosomes purified from glioblastoma cells can uptake synthetic siRNAs targeting Huntingtin mRNA and proficiently carried siRNAs to mouse neurons to bock Huntingtin mRNAs [115]. Ohno and et al. loaded synthetic let-7a miRNAs into HEK293 cells to obtain exosomes encompassing those molecules, thereafter, these exosomes inhibited tumor mass growth in vivo [112]. These results indicate that exosome-based miRNA delivery potentially contributes to tumor regression. However, extra inquiries are essential to uncover fundamental mechanisms related to miRNA signaling pathways in the target cells. Overall, it seems that miRNA therapy is at its infancy and there are still certain limitations regarding miRNA encapsulation into exosomes, the safety of exosomes, and also unwanted results in recipient cells. It is encouraging to declare that there is increasing interest in pre-clinical and clinical applications of miRNAs in specific cancers. By May 2019, the clinical trial data-collection https://clinicaltrials.gov prepared about 52 clinical trials to deal with miRNA ventures (Table 2 and Fig. 2). Scrutiny on the recorded experiments unveiled that the major part of clinical trials belongs to breast cancer diseases (roundly 19.25%). In addition, a study related to using of exosomal miRNAs in prostate cancer patients was recorded by identified number NCT02366494. Taken together, these findings suggest the fact that miRNAs are a new and applicable tool in a clinical situation.

**Table 2:** The list of miRNAs related clinical trials documented up to May 2019

Condition or disease	Type of miRNA	Phase	NCT number
Breast Cancer	Total	Phase-II	NCT02127073
	Total	Phase-IV	NCT01612871
	Total	ND	NCT03779022
	Total	ND	NCT01598285
	Total	ND	NCT02656589
	Total	ND	NCT02065908
	Total	ND	NCT01231386
	Total	ND	NCT01722851
	Total	ND	NCT01957332
	Total	ND	NCT02950207
Prostate Cancer	Total	ND	NCT01220427
	Total	ND	NCT02366494
	Total	ND	NCT02964351
Pancreatic Cancer	Total	Phase-II	NCT02634502
	Total	ND	NCT02531607
	miR-25	ND	NCT03432624
Colorectal Cancer	miR-31-3p and miR-31-5p	Phase-III	NCT03362684
	total	ND	NCT03309722
	Total	ND	NCT02876133
	Total	ND	NCT01712958
Bladder Cancer	miR-155	ND	NCT03591367
Esophageal Cancer	Total	Phase-II	NCT02392377
	Total	ND	NCT02812680
Lung Cancer	Total	ND	NCT02247453
	Total	ND	NCT03452514
	Total	ND	NCT03397355
	Total	ND	NCT03293433
Thyroid Cancer	Total	ND	NCT01964508
	Total	ND	NCT01433809
Brain Tumors	Total	ND	NCT03630861
	Total	ND	NCT01595126
Oral Cancer	miR-21 and miR-200	Phase-III	NCT03685409
	Total	Phase-IV	NCT03684707
	microRNA-29b	ND	NCT02009852
Skin Cancer	Total	ND	NCT00849914
	Total	ND	NCT01143311
	Total	ND	NCT01345760
Kidney Cancer	Total	ND	NCT00806650
Endometrial Cancer	Total	ND	NCT01119573
Ovarian Cancer	Total	ND	NCT02758652
	Total	ND	NCT03738319
Cancer of Head and Neck	microRNA-29 family	ND	NCT01927354
Leukemia	Total	ND	NCT01505699
	microRNA-34a and	ND	NCT01057199
	microRNA-194		
	Total	ND	NCT01229124
	Total	ND	NCT01511575
	Total	ND	NCT00896766
Liver	Total	ND	NCT02448056
	Total	ND	NCT03227510
	miR-221 and miR-222	ND	NCT02928627
	Total	ND	NCT01247506

*ND* Not Determined

### Exosomes as a drug delivery vehicle

**Fig. 3:**
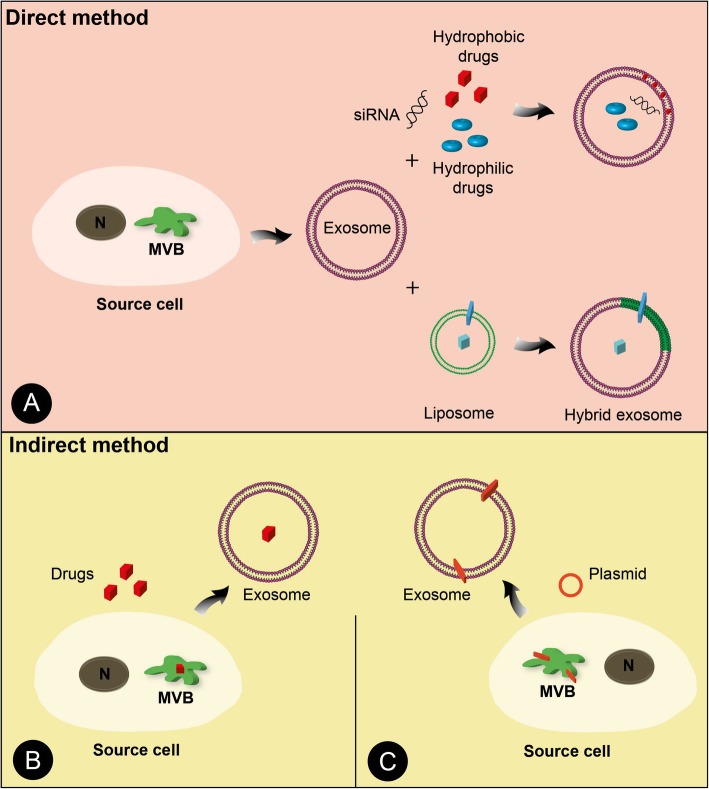
Categories of different methods used to design exosome-based delivery system. In the direct method different compounds such as hydrophilic or hydrophobic drugs and also siRNAs are added to purified exosomes suspension, subsequently, exosomes encompass those compounds. In addition, liposome carrying drugs can be used to construct optional exosome-liposome hybrid vesicles (**a**). In the indirect method parental cells are modified to produce artificial exosomes. In this way, cells co-cultured with different therapeutic agents (**b**) or by using a vector, manipulated to express optional cargoes in exosomes (**c**)

Recently, the approach of nanotechnology has attracted the attention of researchers to the use of pharmacology in medicine. The main purpose of nanoparticle technologies in drug delivery system is the improvement of the efficacy and specificity of therapeutic agents as well as the reduction of their toxicity in the body [116]. In the field of cancer therapy, nanoparticles-based drug delivery system has been approved by the USA Food and Drug Administration (FDA). For example, Doxil® (encapsulated doxorubicin (Dox) into the liposomes) [117] and Abraxane® (paclitaxel (PTX) attached to nanoparticles) are available to use [118]. Interestingly, there is an excessive interest in the use of biological carriers in drug delivery systems that successfully act as a channel to deliver drugs to the target sites. One of these successful candidates is exosome [119]. Exosomes represent one of the major paracrine signaling modules between cells with roles in physiological and pathological conditions, ranging from the normal cell homeostasis to the tumor cells metastasis [120]. A growing number of studies have found that EVs especially exosomes obtained from eligible stem cells can be involved in the treatment of various diseases. For example, the authors showed that exosomes derived from MSCs elicit beneficial effects in disease models [121, 122]. However, several attempts have been made to use purposefully engineered exosomes instead of using unsought exosomes [123]. Thereby, exosomes have attracted scientific attention, because these particles can encapsulate therapeutic agent and efficiently deliver it to target cells [18]. Furthermore, they represent a range of beneficial features such as safety and low immunity, regulating cell signaling, desirable negative zeta potential, the capacity of transferring large amounts of biomolecules, and ability to be engineered for the treatment of various diseases [16, 124]. Furthermore, they can specifically deliver their cargoes to the distantly target sites, even they pass through the blood-brain barrier [16] In this regard, laboratories have examined the possible therapeutic potential of exosomes via sorting RNAs and therapeutic compounds into exosomes in vitro and in pre-clinical experiments. Of note, the exosome-based delivery system has generally designed following two protocols (Fig. 3); I: direct engineering method, by which isolated exosomes directly modified to artificial exosomes and II: indirect engineering method, by which parental cells targeted to modification. According to the clinical trial database (http://clinicaltrials.gov) up to May 2019, when using exosome as a keyword, there are 99 studies about exosomes. To date, only a few exosome-based deliveries clinical trials are listed, therefore, we can point a study in phase I which designed to investigate the delivery of curcumin to colon cancer tissue by plant-derived exosomes (NCT number: 0124072). Additionally, two clinical trials in phase II and I (NCT number: 01159288, NCT number: 03608631) are presented where researchers will engage artificial exosomes against lung and pancreases cancers. In this section, we review exosome engineering methodology and summarize results of exosome therapy in experiments.

### Direct engineering method

Direct engineering method is a promising strategy to load pre-isolated exosomes with therapeutic agents for instance drugs, synthetic compounds, and biomolecules (Fig. 3). Several techniques have been utilized for loading exosome such as simple incubation, electroporation, sonication, extrusion, freeze/thaw cycle, and sponin-assisted methods that summarized in Table 3. In this section, we describe the potential agents directly loaded into exosomes by the different strategies. Recent evidence suggests that exosomes can uptake exogenous compounds in vitro. For example, Zhuang et al. designed an experiment where curcumin incubated with tumor cell-derived exosomes. Consequently, exosomes successfully encapsulated curcumin and delivered it to microglia cells via an intranasal way in a brain inflammatory model. Furthermore, authors successfully encapsulated Stat3 inhibitor into those exosomes [125]. Similarly, curcumin potentially was directed into EL-4 (mouse lymphoma cell line)-derived exosomes, and it has been reported that the efficacy of curcumin was increased by delivering exosomes to inflammatory cells [126]. Using the extrusion method, Fuhrmann and coworkers showed that porphyrins easily were loaded into exosomes collected from different stem cells. Authors demonstrated that artificial exosomes produced by the extrusion method show significant anticancer property compared with exosomes prepared via a passive method (incubation) [127]. Researchers also examined the encapsulation of chemotherapy drugs into exosomes. For instance, it has been shown that exosomes isolated from mouse immature DCs are capable of loading chemotherapeutics drugs like Dox by electroporation technique, and they specifically deliver the drug to the tumor tissue in BALB/c nude mice [19]. The ability of PTX to occupy exosome lumen confirmed by Kim et al. experiment in which PTX loaded into macrophages-derived exosomes by sonication method; resulted in amplified cytotoxicity response in the drug-resistant cancer cells [128]. Researchers have also attempted to encapsulate biomolecules into isolated exosomes. For instance, Alvarez-Erviti and colleagues showed that exosomes encapsulated exogenous siRNAs into their lumen via electroporation method. Additionally, the authors reported that these exosomes intravenously delivered siRNAs to neuron and glial cells in the brain and led to gene inhibition [129]. Thereafter, recent studies have found that exogenous siRNAs could be internalized into exosomes in different ways and may cause gene knockdown in target cells [130, 131]. It has recently been shown that glioblastoma-derived exosomes successfully uptake modified siRNAs targeting Huntingtin mRNA and efficiently delivered siRNAs to mouse neurons and led to silence Huntingtin mRNAs [115]. Similarly, in a recent work, using sonication or electroporation, purified exosomes from HEK293 and MCF-7 cells encompassed exogenous siRNAs, miRNA, and single-stranded DNA (ssDNA) oligonucleotides. These exosomes have been confirmed that induced anticancer characteristics through silencing HER2 genes [132]. Another interesting study evaluated the capacity loading of DNA molecules into exosomes. Lamichhane et al. used electroporation method that linear DNA was sorted into exosomes. They also revealed that there is a direct relationship between DNA loading efficiency and size of linear DNA molecules [133]. Haney et al. conducted an interesting experiment where attempted to load catalase into exosome ex vivo. Using exosomes from macrophages, authors tested the efficacy of different loading strategies such as incubation, extrusion, freeze/thaw cycle, sonication, and even saponin-assisted to sort catalase into exosome. Exosomes from diverse source differently encapsulated catalase and efficiently delivered it to neuronal cells in vitro and into the brain of Parkinson’s disease mouse model and showed considerable neuroprotective effects [134]. Other compounds such as dextran molecules were successfully loaded into exosomes [135]. Collectively, the direct engineering method seems to be a promising strategy to sort specific compound into exosomes. In addition, however, further studies are necessary to elucidate which loading method has high efficacy and acts inclusively at the same time.

**Table 3:** Comparison of the methods utilized for loading of therapeutic agents into exosomes

Methods	Therapeutic agents	Advantage	limits
Incubation	Paclitaxel/Curcumin, siRNAs, Porphyrins, Catalase, Doxirubicin	simple	Low loading efficiency Drugs may harm cells
Electroporation	Doxirubicin, siRNAs, linear DNA, Catalase	large molecules can be loaded	Low loading efficiency, Interrupts exosome integrity, siRNA clump
Extrusion	Porphyrins, Catalase	High drug loading efficiency	Alternations in membrane
Sonication	Paclitaxel, siRNAs, Catalase,	High drug loading efficiency, Suitable for small RNAs	Alternations in membrane Not suitable for hydrophobic agents
Freeze/thaw	Catalase, DOX	Potential of membranes fusion	Low loading Efficiency Cramped exosomes
Saponin-assisted	Catalase, Porphyrins, DOX	Relative high drug loading efficiency	Toxicity and harmful effects long protocol

### Indirect engineering method

#### Drug loading

In this protocol, therapeutic agents such as drugs exogenously packed into parental cells (Fig. 3). In the case of drug loading techniques, cells are incubated with drugs in vitro, and after the incubation period, purified exosomes can be subjected to down-stream studies (Fig. 3). Pioneering research by LV et al. examined the effect of anticancer drugs on exosomal HSPs protein loading potential in hepatocellular carcinoma cells (HepG2) in vitro. Authors showed that anticancer drugs simultaneously increased exosome secretion and altered HSPs content in isolated exosomes. Interestingly, these exosomes powerfully motivated NK cells cytotoxicity, therefore induced immunogenicity responses in NK cells [136]. It has been proven that PTX, an anticancer chemotherapy drug when co-cultured with MSCs over 24 h; it could enter into EVs. Interestingly, PTX-bearing EVs exhibited more anticancer effect in comparison with EVs derived from the control group [137]. In addition, the liposome-mediated method was also used to construct artificial EVs, that using this method hydrophobic compounds filled into EVs [138]. In line with this, a similar work showed that lipophilic and hydrophilic drugs could be co-sorted powerfully into the purified EVs when their parental cells treated with membrane fusogenic liposomes containing those drugs [139].

#### Parental cell modification

One of the exciting methods is the genetically manipulating of parental cells to produce artificial exosomes (Fig. 3). In this manner, there is an interesting approach to using the exosome membrane as a therapeutic tool. Indeed exosome surface molecules engineered to enhance efficiency and sensitivity of the exosome-based delivery system [19]. Besides this, parental cells engineered to generate optional exosomes containing specific biomolecules for deliver cargoes to target cells; in fact, exosomes designed to carry specific molecules. According to this statement, DCs were transfected to over-express Lamp2b, located in the exosomal membrane, and combined to the neural RVG peptide to produce optional exosomes with a modified membrane which bind specifically to neuron cells in Alzheimer’s disease mouse model [129]. In a study which set out to modify exosomes membrane, Liu et al. found that cells over-expressing of RVG-Lamp2b peptide produced exosomes containing RVG-Lamp2b peptide on the membrane surface. These exosomes specifically delivered opioid receptor mu (MOR) siRNA to the mouse brain and Neuro2A cells in vitro. Of note, concurrently, the MOR siRNA oligonucleotides transfected to cells to produce exosomes bearing those siRNAs [140]. Ohno and et al. designed a wonderful experiment by which HEK293 modified to over-express GE11 or EGF molecules. They collected exosomes from CM and confirmed the expression of GE11 or EGF proteins on the exosome membrane. After that, the delivery capacity of exosomes in an in vitro and also in xenograft breast cancer tissue in RAG2−/− model was proved. Simultaneously, they showed that exosomes were strongly trapped in an EGFR dependent manner and synthetic let-7a miRNAs subjected to tumor mass in vivo [112]. Authors indicated that artificial exosomes are capable of delivering therapeutic agents to EGFR-expressing tumors. As mentioned above; some scientists manipulated stem cells to produce exosomes with optional cargoes for the treatment of diseases. For instance, Gnecchi and colleagues showed that CM from MSCs -Akt-overexpressing strongly improved cardiac function in an infarcted mouse model [141]. Furthermore, U87MG glioblastoma cells were manipulated to become overexpressing- Delta-like 4 (Dll4) cells which abundantly produced exosomes-bearing DII4. Purified exosomes-bearing DII4 efficiently were capable of increasing angiogenesis both in vitro and in vivo models [142]. It was shown that exosomes from human CD34^+^-Shh-overexpressing cells transfer Shh molecules to ischemic myocardium and stimulate the Shh signaling pathway in target cells [143]. In a murine collagen-induced arthritis experiment, DCs were genetically transfected with indoleamine 2, 3-dioxygenase (IDO) to produce optional exosomes. Authors declared that exosomes carrying IDO exhibit immunosuppressive and anti-inflammatory effect and inhibit arthritis [144]. In a study, THP-1 macrophages transfected with anti-oncomirs such as miR-143 that cells successfully exported miR-143 molecules associated with exosomes. These exosomes exhibited antitumor effects [145]. Furthermore, there has been an interest in a method by which stem cells are being activated to produce functional secretomes. For instance, Salomon et al. cultured umbilical derived MSCs under hypoxia and examined the angiogenic potential of their exosomes in vitro. The result showed that oxygen stress elevated exosome secretion rate and hypoxic exosomes significantly encouraged angiogenesis [146]. In a similar vein, exosomes derived from a hypoxic human leukemia cell line (K562) profoundly increased angiogenesis in endothelial cells compared to exosomes from normoxic k562 cells. Further scrutiny revealed that hypoxic exosomes abundantly contain miRNA-210 which target angiogenesis signaling pathway [147]. Angiogenic potential of exosomes isolated from activated THP-1, a human monocyte cell line, has also been observed. THP-1 cell line was activated by different inflammatory factors which resulted in an increased level of miRNA-150 in exosomes, subsequently, exosomes enhanced endothelial cell migration [110]. Despite the few studies, these experiments offered thus far present evidence that parental cell activation may be a useful strategy to produce functional exosomes.

## Conclusion

In recent years, the significance of exosomes as intercellular mediators has been described. Tumoral exosomes by transferring miRNAs play pivotal roles in tumor maintenance. As tumoral exosomes contain tumor information and also available in almost bio-fluids, exosomes can provide a novel approach in early diagnosis of cancers and serve as a tumor biomarker. In addition, modification of exosomes may able researchers to deliver tumor-suppressive miRNAs to tumor cells. However, this is required to determine exactly how miRNA were encapsulated into exosomes and which miRNAs are suitable to use as a tumor inhibitor and cancer biomarker. In general, the exosome-based delivery vehicle represents an efficient system by which a large number of materials can be purposely delivered to target sites in the body. The advent of safe nano-carriers with high efficacy is the main goal of nano-medicine. Thus, the advance of artificial exosomes has opened a hopeful opportunity for the delivery of therapeutic agents. Nonetheless, future studies on the present issue are therefore recommended answering some questions; which cells should be manipulated to produce optional exosomes? In another word, which exosomes are safe and bio-compatible for drug delivery system? And, What is the suitable and nontoxic method for the modification of exosomes? In addition, the majority of experiments are carried out in vitro and ultimately in vivo models, therefore, the safety, specificity, and efficiency of this system in clinical trials remains still more elusive.

## Data Availability

Not applicable

## Abbreviations

ABs: Apoptotic Bodies
Ago2: Argonaute2
CM: Conditioned Medium CRC: colorectal cancer
DCs: Dendritic Cells
Dll4: Delta-like 4
Dox: Doxorubicin
ECM: Extracellular Matrix
ECs: Endothelial Cells
ESCRT: Endosomal Sorting Complex Required For Transport
EVs: Extracellular Vesicles
FDA: USA Food and Drug Administration
HEK293: Human Embryonic Kidney Cell Line 293
HepG2: Hepatocellular Carcinoma Cells
HuR: Human Antigen R
ICAM-1: Adhesion Molecule 1
IDO: Indoleamine 2,3-dioxygenase
ISEV: International Society for Extracellular Vesicles
miRNAs: MicroRNAs
MMPs: Matrix metalloproteinase
MOR: Opioid Receptor Mu
MSCs: Mesenchymal Stem Cells
MVBs: Multivesicular Bodies
nSMase2: Sphingomyelinase 2
PM: Plasma Membrane
PTX: Paclitaxel
RBPs: RNA-binding proteins
RFXAP: Regulatory factor X-associated protein
RLC: RISC-Loading Complex
rRNA: Ribosomal RNA
SNAREs: Soluble NSF attachment protein receptors
ssDNA: Single-stranded DNA
TRL: Toll-like Receptors
TSAP6: Tumor suppressor activated pathway 6
